# Preparation of B_4_C_p_/Al Composites via Selective Laser Melting and Their Tribological Properties

**DOI:** 10.3390/ma15238340

**Published:** 2022-11-23

**Authors:** Guodong Yang, Jialian Zhang, Houbo Xie, Faliang Li, Zhong Huang, Gaoqian Yuan, Jingzhe Zhang, Quanli Jia, Haijun Zhang, Hasibe Aygul Yeprem, Shaowei Zhang

**Affiliations:** 1 The State Key Laboratory of Refractories and Metallurgy, Wuhan University of Science and Technology, Wuhan 430081, China; 2 Henan Key Laboratory of High Temperature Functional Ceramics, Zhengzhou University, Zhengzhou 450052, China; 3 Department of Metallurgy and Materials Engineering, Yildiz Technical University, Istanbul 34349, Turkey; 4 College of Engineering, Mathematics and Physical Sciences, University of Exeter, Exeter EX4 4QF, UK

**Keywords:** selective laser melting, B_4_C_p_/Al composites, scanning speed, relative density, tribological property

## Abstract

B_4_C-particle-reinforced Al (B_4_C_p_/Al) composites are widely used in various areas, e.g., armors, electronic packaging and fuel storage, owing to their several outstanding properties including high specific rigidity, excellent wear resistance and light weight. Selective laser melting (SLM) is favored in manufacturing complex components because of its high raw material utilization rate and high efficiency. In this work, a B_4_C_p_/Al composite was successfully synthesized by SLM, and the effects of one of the most important parameters, scanning speed (100–700 mm/s), on the phase composition, density, microhardness and tribological properties of the samples were investigated. The microhardness, relative density and dry-sliding wear resistance of as-prepared B_4_C_p_/Al composites were improved with the decrease in scanning speed, and the sample fabricated at a scanning speed of 100 mm/s exhibited a relative density as high as about 97.1%, and a maximum microhardness of ~180 HV_0.1_ (approximately six times more than that of the SLM-formed pure Al sample, 31 HV_0.1_), a minimum wear rate of 4.2 × 10^−5^ mm^3^·N^−1^·m^−1^ and a corresponding friction coefficient of 0.41. In addition, abrasive wear, adhesive wear and oxidation wear were found to be behind the overall wear behavior of as-prepared B_4_C_p_/Al composites.

## 1. Introduction

B_4_C particles are often used as reinforcements to prepare various composites because of their excellent physical and chemical properties including high hardness, good wear resistance and strong chemical stability [1,2]. Because of their high specific stiffness, light weight, good toughness and plasticity, good corrosion resistance and excellent wear resistance [3], B_4_C_p_/Al composites have attracted much attention from researchers and been successfully used for vehicle and body armors, aerospace structures, electronic packaging, fuel storage and nuclear radiation protection [4]. To fabricate such composites of high quality, various methods including stir-casting, squeeze-casting, pressureless infiltration and ultrasound-assisted casting have been employed [5,6,7,8,9,10]. However, they suffer from various drawbacks such as severe phase segregation, poor interfacial bonding, high cost, low efficiency and difficulty in manufacturing a component of a complex geometry.

As a major additive manufacturing technique, selective laser melting (SLM) features the direct fabrication of three-dimensional (3D) parts with complicated structures [11]. Compared with the conventional manufacturing methods listed above, SLM used in preparing B_4_C_p_/Al composites with complex structures shows many advantages including high precision, high utilization rate of raw materials, high applicability and remarkable mechanical properties of the manufactured products [12]. It was adopted in the preparation of various metal and alloy materials, for example titanium-, nickel- [13], iron- [14] and aluminum-based [15] materials for aerospace, automotive, military, nuclear power, shipbuilding and medical applications. For instance, the yield strength and tensile strength of Al-12Si alloys fabricated by SLM reached 260 MPa and 380 MPa, respectively, which were four times and twice as high, respectively, as that of their counterparts prepared by a casting method [16], and Al_2_O_3_/Al composites prepared by SLM exhibited a continuous and compatible interface between the Al_2_O_3_ and Al matrix and showed very high hardness and wear performances [17].

The laser scanning speed [18] has been proven to be important in the SLM preparation of composite materials. Ni et al. [19] fabricated 316L stainless steel via SLM and discussed the effect of the scanning speed on its corrosion resistance. They found that the number of voids and corrosion increased with increasing the scanning speed. Lu et al. [20] reported that the density, yield strength and corrosion resistance of SLM-fabricated Ni-free CoCrW materials were mainly determined by the laser scanning speed. Matras et al. [21] pointed out that an increase in the laser scanning speed caused deterioration to a certain extent in the surface roughness of SLM-manufactured AlSi10Mg semi-finished parts. Sadali et al. [22] observed that micro-cracks increased in as-prepared Ti6Al4V parts with the scanning speed, reducing the splashing effect of raw materials during SLM.

Based on the above discussions, in the present work, B_4_C_p_/Al composites were further fabricated by SLM, and the effects of the scanning speed on their density, phase composition, microstructure, interfacial bonding, microhardness and tribological properties were examined in detail.

## 2. Experimental Procedure

### 2.1. Raw Material Powder

**Figure 1:**
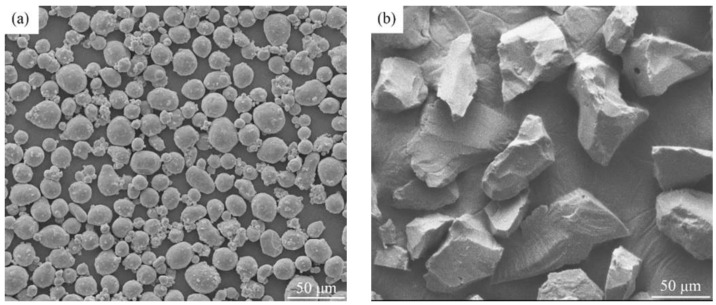
Microstructural images of the starting powders of Al (**a**) and B_4_C (**b**).

Spherical Al powder (purity 99.3%, mean diameter of 33 μm, Figure 1a) and angular B_4_C particles (purity 99.8%, average size of 65 μm, Figure 1b) were used as raw materials. They were mixed in a weight ratio of 4:1 for 12 h in a DECO-PM-2*5L ball mill with a rotating speed of 10 rpm and ball-to-powder weight ratio of 1:1.

### 2.2. SLM Processing

**Figure 2:**
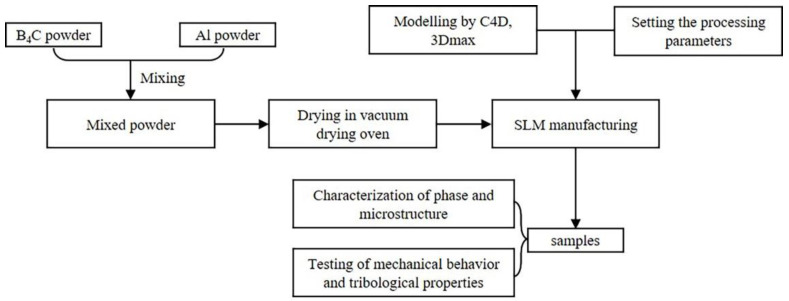
Flow chart of the overall experimental procedure.

All the samples were fabricated on stainless steel substrates by a WJ SLM225 device with a YLR-500-WC-fiber laser under the protection of high-purity argon gas. To investigate the effect of the scanning speed on the preparation of B_4_C_p_/Al composites, a series of samples with dimensions of 10 × 10 × 5 mm^3^ were prepared by using a laser beam of 1.07 μm in wavelength and 100 μm in spot size to scan the starting powders at different speeds (100, 300, 500 and 700 mm/s) while fixing the following processing parameters: 0.05 mm scanning distance, 0.05 mm layer thickness and 250 W laser power. A laser scanning mode with a rotation of 17° between neighboring layers was applied for minimizing the thermal stress formed during the SLM process, and the main processing parameters are listed in Table 1. The detailed SLM procedure has already been reported in our previous work, and is shown in Figure 2 [23].

### 2.3. Phase and Microstructure

The SLM-formed samples cut off from the substrates were ground and polished following the standard procedure and subjected to 40 s chemical etching with the Kroll’s reagent (95 vol.% of H_2_O, 1 vol.% of HF, 1.5 vol.% of HCl and 2.5 vol.% of HNO_3_) at room temperature prior to the following examination.

Phases of the samples were determined by X-ray diffraction (XRD, X’pert pro, Philips, The Netherlands) operating at 60 mA and 60 kV with Cu *Kα* radiation (*λ* = 0.15406 nm). The theoretical density (D_T_) was calculated from the actual volume fraction of each phase, and the bulk density (D_B_) was measured by using the Archimedes’ method, from which the relative density (D_R_) of the samples was further calculated (D_R_ = D_B_/D_T_). Phase morphologies and elemental compositions of the samples were examined on an FEI Nova NanoSEM 400 scanning electron microscope (SEM, FEI, Hillsboro, OR, USA) equipped with a Penta FET X-3 Si (Li) energy-dispersive spectrometer (EDS).

### 2.4. Mechanical Behaviour

Microhardness values of the samples were determined on a Vickers hardness tester (SH-318-III equipment) under a 100 g load for 10 s from multiple points on their cross section, and the average was recorded. The wear behavior and friction at room temperature were examined by a UMT-2 machine and wear-testing machine via the reciprocating test of ball-on-flat under the following conditions: dry friction, 20 N normal load, 5 mm/s friction speed, 6 mm friction stroke and 20 min duration. Carbon steel balls (HRC62) of 2 mm in diameter were used for the linear reciprocating motion. Each sample was tested three times under identical conditions, and the average values of the friction coefficient and wear rate were calculated.

## 3. Results and Discussion

**Figure 3:**
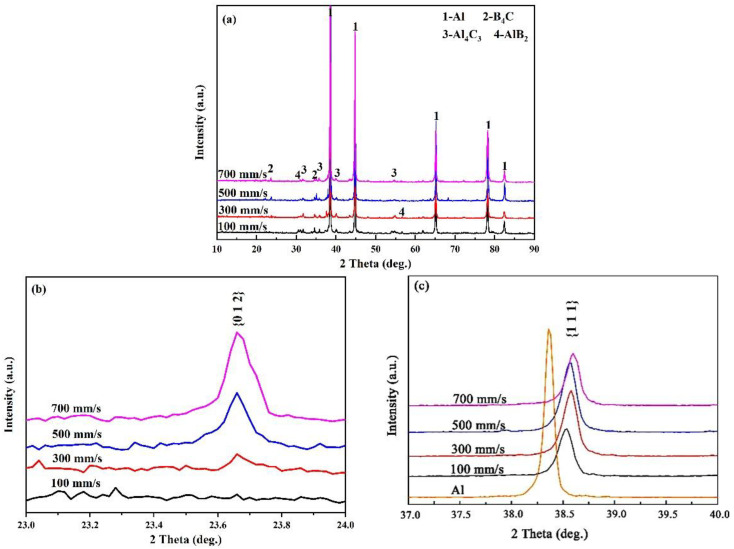
XRD patterns of B_4_C_p_/Al composites fabricated via SLM at different scanning speeds: (**a**) 2*θ* = 10–90°; (**b**) 2*θ* = 23–24°; (**c**) 2*θ* = 37–40°. (Al: ICCD 064700; B_4_C: ICCD 029093; Al_4_C_3_: ICCD 014397; AlB_2_: ICCD 043851).

Shown in Figure 3a are the XRD patterns of the B_4_C_p_/Al composite samples prepared with various scanning speeds, revealing the coexistence of Al and B_4_C in them. Apart from these, Al_4_C_3_ and AlB_2_ were detected in all the cases, suggesting the in situ reaction between the Al melt and B_4_C. AlB_2_ and Al_4_C_3_ can be formed at 898–963 K and 1423–1458 K, respectively, via the reaction of B_4_C with Al melt [24], implying that the temperature of the mixed powders during the SLM process reached above 1423 K. The diffraction peak of the B_4_C phase at 2*θ* = 23.66° (Figure 3b) increased with the laser scanning speed, since a lower scanning speed provided a higher bulk energy density, favoring the increase in the molten pool temperature and thus promoting the reaction between the Al melt and B_4_C [23,25]. Figure 3c illustrates that the {111} diffraction peak of Al shifted to the right with respect to that of the starting Al powder, demonstrating that certain extents of lattice distortion occurred due to the laser-induced non-equilibrium [26]. Moreover, the intrinsic characteristic of the rapid heating/cooling rate during the SLM processing resulted in residual stress in the sample, which further intensified the lattice distortion [27].

**Figure 4:**
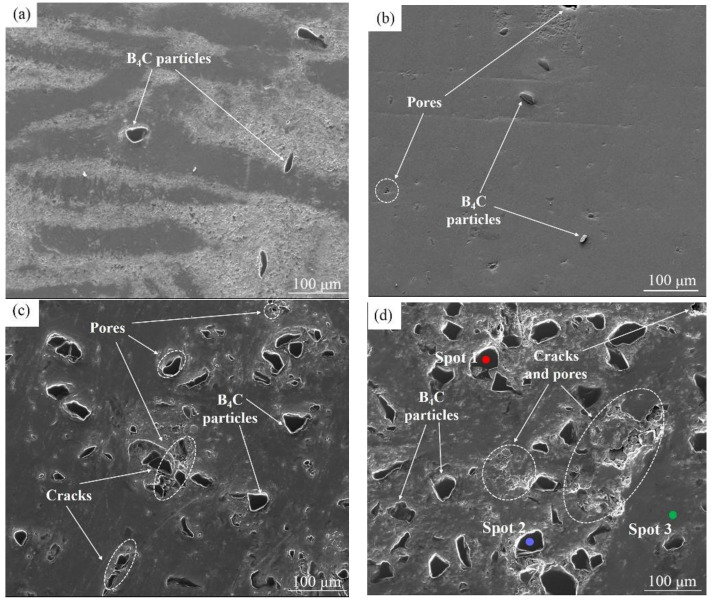
Cross-sectional SEM images of B_4_C_p_/Al composite samples prepared at different laser scanning speeds: (**a**) 100 mm/s; (**b**) 300 mm/s; (**c**) 500 mm/s; (**d**) 700 mm/s.

**Figure 5:**
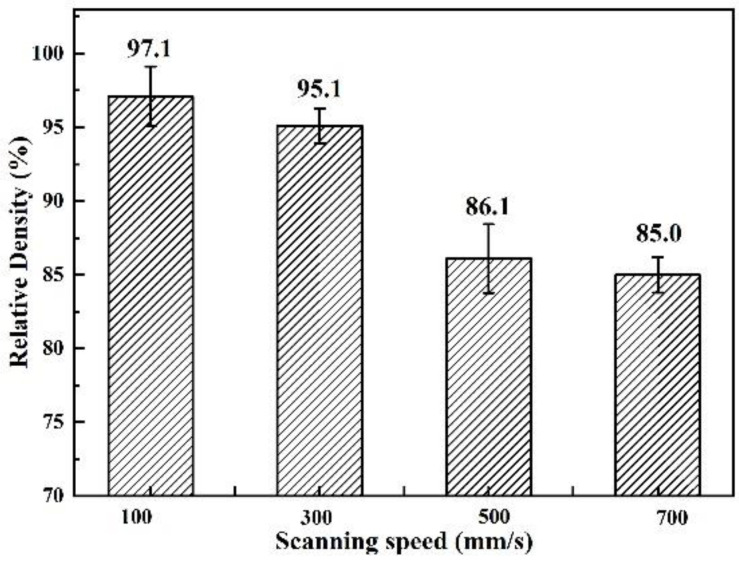
Relative density values of B_4_C_p_/Al composite samples as a function of laser scanning speed.

Figure 4 and Figure 5 show the cross-sectional SEM images of B_4_C_p_/Al composites prepared at different laser scanning speeds and their relative density values, respectively. When the laser power, layer thickness and hatch space were given, the increase in the laser scanning speed led to a decrease in the input energy, hindering the formation and fluidity increase of the Al melt. Since the temperature of the molten pool at a high laser scanning speed was low, “larger”-sized B_4_C particles were observed in the sample (Figure 4d, Table 2). Furthermore, the limited formation and low fluidity of the Al melt at a high laser scanning speed delayed the densification of the B_4_C_p_/Al composites, resulting in more defects (pores and cracks) in them (Figure 4a–d). Correspondingly to Figure 4a–d, the density of the samples decreased from 97.1% to 85% when the scanning speed increased from 100 to 700 mm/s (Figure 5).

Similarly to the case of SiC_p_/Al composite preparation, the SLM process in the present case is also believed to be dominated by the complete melting–solidification mechanism [23]. Initially, a molten pool was formed under the laser irradiation. Then, the high temperature melt with good fluidity flowed around to fill various forms of voids, following which an in-situ reaction between the melt and nonmelted particles occurred. The temperature of the formed molten pool played a major role in the densification of the B_4_C_p_/Al composite samples. Compared with B_4_C_p_, Al has a much lower melting point and a stronger laser absorption ability [28]; therefore, the Al powders absorbed the laser energy and melted to form the molten pool. The temperature in the molten pool can be calculated by the following Equation (1) [29]:

(1) $$\Delta T_{max}=2\;A\;\eta (\sqrt{\frac{k_{th}\;\tau_{p}}{\pi }})/k$$

In Equation (1), *A*, *η*, *k_th_*, *τ_p_* and *k* represent the laser absorptivity, volume energy density, heat diffusivity, time duration and thermal conductivity, respectively. After these parameters are determined, the temperature change (Δ*T_max_*) in the laser-induced molten pool can be calculated according to Equation (1). Δ*T_max_* is directly proportional to the energy density (*η*), which is determined by the following Equation (2) [30]:

*η* = *P*/(*v* ∙ *h* ∙ *d*) (2)

where *P* represents the laser power, *v* the scanning speed, *h* the hatch space and *d* the layer thickness.

According to Equation (2), the energy density is inversely proportional to the scanning speed. When other processing parameters are given, as indicated by Equations (1) and (2), a high energy density arising from a low scanning speed led to an increase in the temperature of the powder bed, facilitating the formation of a liquid phase and the subsequent densification of the samples.

**Figure 6:**
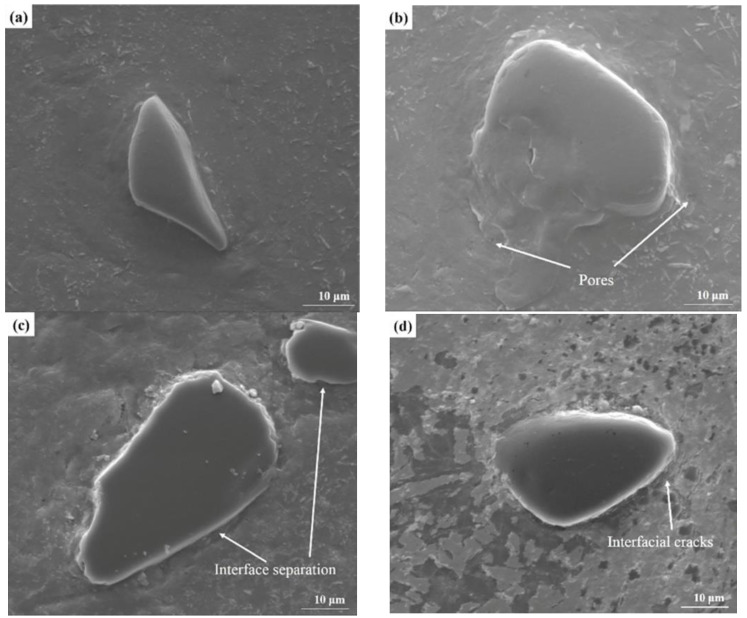
SEM images of B_4_C_p_/Al composite samples fabricated via SLM at different scanning speeds: (**a**) 100 mm/s; (**b**) 300 mm/s; (**c**) 500 mm/s; (**d**) 700 mm/s. The images highlight the representative interfaces between B_4_C_p_ and Al.

Presented in Figure 6 are SEM images of representative interfaces between B_4_C particles and the Al matrix in the B_4_C_p_/Al composites fabricated at different scanning speeds. In the case of using a low scanning speed, the B_4_C particles were in close contact with the surrounding Al matrix, and less defects were present at their interface (Figure 6a–b). On the other hand, in the case of using a high scanning speed, some pores and micro-cracks were seen at the interface (Figure 6c–d). Strong interfacial bonding was beneficial to the enhancement of the physical properties of the as-fabricated composite samples [31,32].

**Figure 7:**
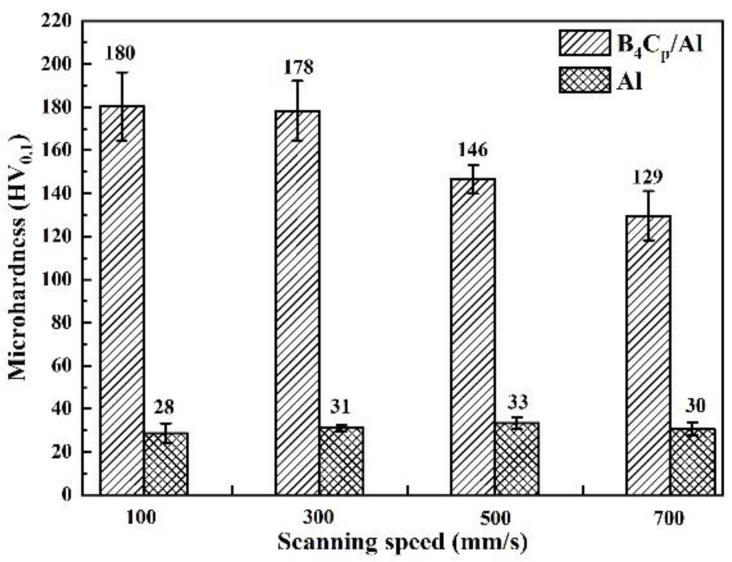
Microhardness values of B_4_C_p_/Al composite and Al alloy samples formed by SLM at different scanning speeds.

Figure 7 gives microhardness values of the B_4_C_p_/Al composite and Al alloy samples prepared via SLM, revealing the negative effect of the high scanning speed on the microhardness. With the increase in the scanning speed from 100 to 700 mm/s, the microhardness of the composite samples decreased from 180 HV_0.1_ to 129 HV_0.1_. This was because with decreasing the scanning speed, the relative density of the samples increased (Figure 5) and their defects (pores and micro-cracks) decreased. Surprisingly, the maximum microhardness value of the as-formed B_4_C_p_/Al composite samples (180 HV_0.1_) was about six times as high as that of the Al alloy sample (31 HV_0.1_), demonstrating the great effect of B_4_C particle reinforcement on the microhardness of the Al matrix. Similar effects were also found in the cases of SiC_p_/Al [23,33], (TiC + TiB_2_)/Al [34], SiC_p_/A365 [35] and TiC/Ni [36] composites prepared via SLM.

**Figure 8:**
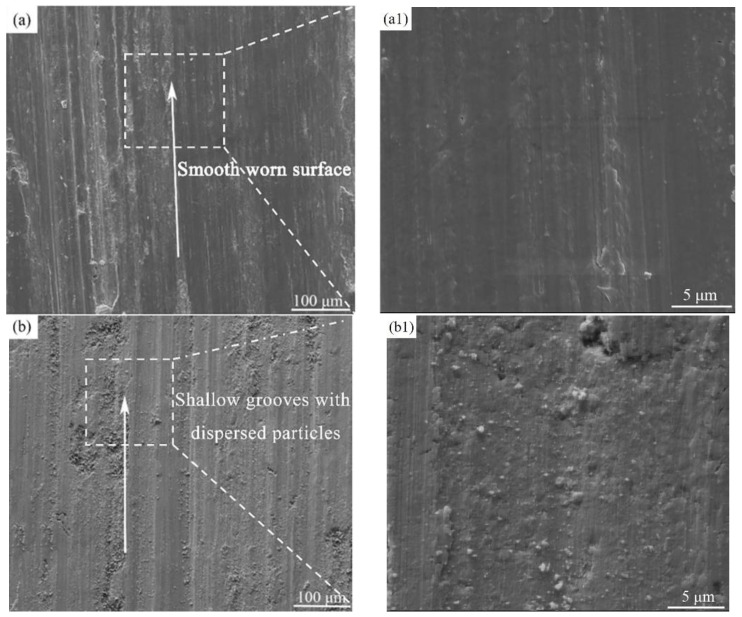

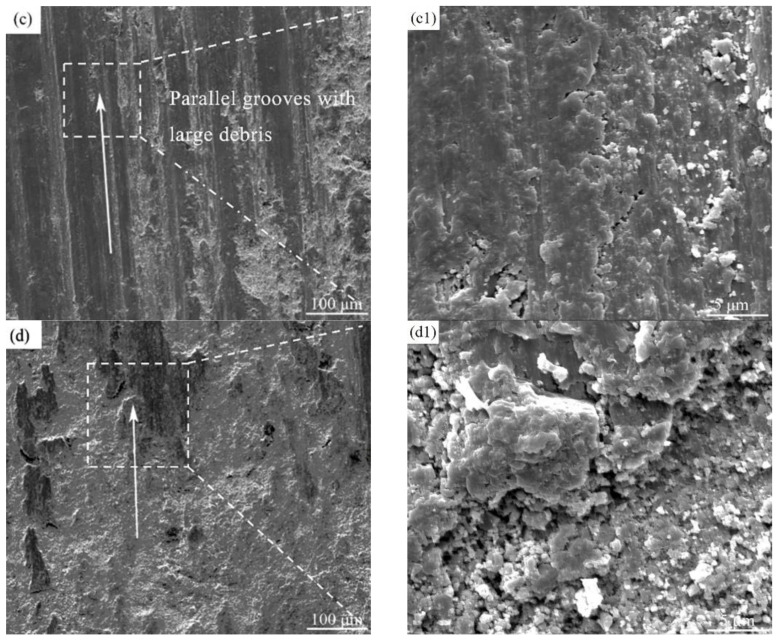
Low- (**left column**) and high-magnification (**right column**) SEM images of worn surfaces of SLM-prepared B_4_C_p_/Al samples corresponding to different scanning speeds: (**a**,**a1**) 100 mm/s; (**b**,**b1**) 300 mm/s; (**c**,**c1**) 500 mm/s; (**d**,**d1**) 700 mm/s.

Figure 8 shows low- and high-magnification SEM images of wear surfaces of the B_4_C_p_/Al samples corresponding to different scanning speeds. At a scanning speed as low as 100 mm/s, only shallow furrows with rough edges were observed on the worn surface (Figure 8a), indicating that the abrasive wear mechanism dominated the wear process of the B_4_C_p_/Al composite sample. At a higher scanning speed of 300 mm/s, a few abrasive particles besides furrows were adhered to the worn surface (Figure 8b), indicating the dominance of the abrasive–adhesion wear mechanism. On increasing the scanning speed to 500 mm/s, some peeling-off debris and deeper furrows were observed (Figure 8c), revealing the dominance of the adhesive wear mechanism. On further increasing the scanning speed to 700 mm/s, the peeling-off, as expected, resulted in lots of wear debris (Figure 8d), confirming the governing of the adhesive wear mechanism. Overall, more defects including furrows, abrasive particles and debris were formed on the worn surface as the scanning speed increased, which was due to the reduced densification and weakened interfacial bonding between B_4_C particles and the Al matrix of the composite samples [23].

**Figure 9:**
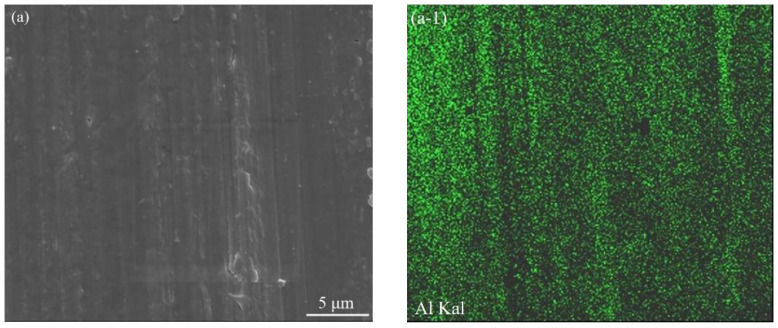

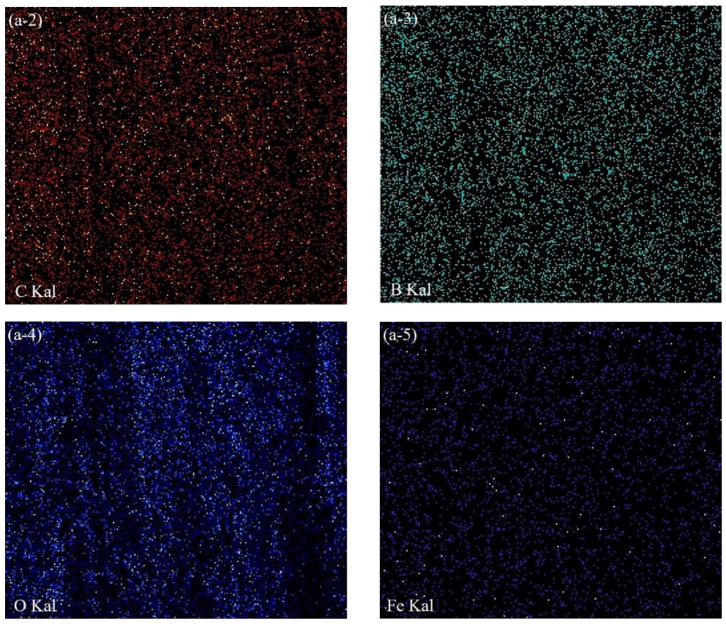
EDS mappings of worn surface of the SLM-formed B_4_C_p_/Al sample prepared at a scanning speed of 100 mm/s: (**a**) SEM image of examined area; and elemental distributions of (**a-1**) Al, (**a-2**) C, (**a-3**) B, (**a-4**) O and (**a-5**) Fe.

To further explore the wear mechanism of the as-fabricated B_4_C_p_/Al composites, the worn surface was additionally examined by high-magnification SEM. As presented in Figure 9, not only Al, C and B elements but also O and Fe elements were detected on the worn surface of the sample fabricated at a scanning speed of 100 mm/s, suggesting the occurrence of additional oxidative wear, and tribo-oxides generally play a positive role in reducing the wear [37]. The presence of Fe implied a possible change in the grinding medium (HRC62 carbon-steel balls). For the other composite samples prepared under higher scanning speeds, the element distributions were similar (no further description here to avoid repetition).

**Figure 10:**
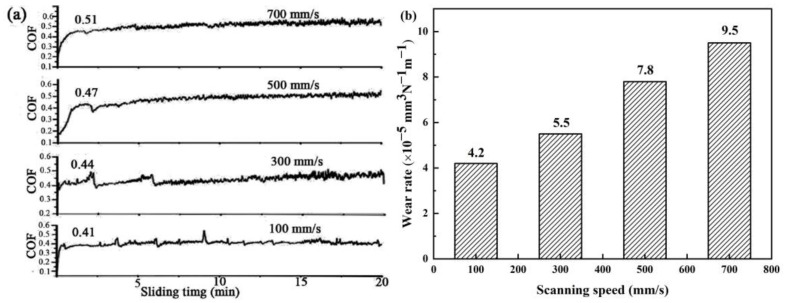
Effects of scanning speed on coefficients of friction (COF) (**a**) and wear rate (**b**) of B_4_C_p_/Al samples.

Figure 10 demonstrates the relationships between the laser scanning speed, the wear rate of the B_4_C_p_/Al sample and the corresponding friction coefficient, revealing that the latter two increased with increasing the first one. As the scanning speed increased from 100 mm/s to 300, 500 and 700 mm/s, the friction coefficient was increased from 0.41 to 0.44, 0.47 and 0.51, respectively. Meanwhile, the wear rate was increased from 4.2 × 10^−5^ to 5.5 × 10^−5^, 7.8 × 10^−5^ and 9.5 × 10^−5^ mm^3^·N^−1^·m^−1^, respectively. These results could be attributed to the higher density, stronger interfacial bonding and higher microhardness of the samples prepared at a lower scanning speed (Figure 4, Figure 5, Figure 6 and Figure 7).

**Figure 11:**
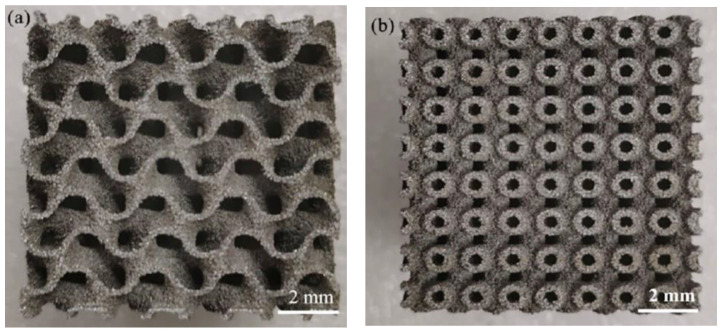
Two representative components (**a**,**b**) with complex matrix structures fabricated by SLM.

In addition to the samples described above, some representative components with complex geometries (Figure 11a,b) were successfully fabricated by SLM using B_4_C particles and Al powder as raw materials, further demonstrating the universal feasibility of the SLM technique in preparing an Al matrix of a complex structure. To compare, Table 3 lists the tribological and other mechanical properties of B_4_C-particle-reinforced Al matrix composites prepared by other methods, revealing the higher microhardness and superior tribological property of the B_4_C_p_/Al composite samples prepared in the present work.

## 4. Conclusions

A B_4_C_p_/Al composite with considerably improved mechanical properties was successfully prepared via SLM. The main conclusions are drawn as follows:

(1) The densification of the SLM-prepared B_4_C_p_/Al composite samples decreased from 97.1% to 85% with increasing the scanning speed due to the decreased molten pool temperature, resulting in a weakened interfacial bonding between the B_4_C reinforcement phase and the Al matrix.

(2) The increase in the scanning speed showed negative effects on the tribological properties and microhardness of the B_4_C_p_/Al composite samples due to the reduced relative density. The composite sample prepared with the lowest scanning speed exhibited the maximum microhardness of 180 HV_0.1_, minimum wear rate of 4.2 × 10^–5^ mm^3^·N^−1^·m^−1^ and a corresponding friction coefficient of 0.41. Encouragingly, the maximum microhardness of the B_4_C_p_/Al composite sample prepared in this work was six times as high as that of the Al alloy counterpart.

(3) The wear mechanism of as-prepared B_4_C_p_/Al samples changed from abrasive wear to adhesive wear with increasing the scanning speed. Additionally, oxidation wear was also involved in the wear process.

## Figures and Tables

**Table 1:** SLM processing parameters.

No.	Laser Power [W]	Scanning Speed [mm/s]	Hatch Space [mm]	Layer Thickness [mm]	Energy Density [J/mm^3^]
1	250	100	0.05	0.05	1000
2		300			333
3		500			200
4		700			143

**Table 2:** EDS analysis of spots 1, 2 and 3 in Figure 4d.

Spot No.	Atomic Content/%			
	B	C	Al	O
Spot 1	79.3	18.2	2.5	0
Spot 2	78.5	19.0	2.5	0
Spot 3	0	0	89.1	10.9

**Table 3:** Tribological and other mechanical properties of B_4_C-particle-reinforced Al matrix composites reported previously.

Method	Matrix	B_4_C Fraction	Relative Density	Hardness	Wear Rate	Friction Coefficient	Ref.
Post densification heat treatment	Pure Al	70 vol.%	N/A	81 HRA	N/A	N/A	[38]
Hot-press infiltrate	6061Al alloy	30 wt.%	98%	123.6 HV_0.5_	N/A	N/A	[39]
Spark plasma sintering	6061Al alloy	20 vol.%	99.32%	146 HV_1_	N/A	N/A	[40]
Powder metallurgy technique	6061Al alloy	20 wt.%	N/A	40 HRB	N/A	N/A	[41]
Casting technique	AA7075 alloy	10 vol.%	N/A	175 BHN	7 × 10^−6^ g/m	0.327	[42]
Stir-casting technique	A356 alloy	10 vol.%	N/A	N/A	2.1 × 10^−3^ mg/m	N/A	[43]
Stir-casting technique	AA2014 alloy	12 vol.%	91.75%	107 BHN	N/A	N/A	[44]
Hot isostatic pressing	AA5083 alloy	10 wt.%	N/A	N/A	N/A	0.41	[45]
Squeeze-casting technique	Pure Al	10 vol.%	N/A	51 ± 3 HV_5_	N/A	0.31	[46]
Stir-casting technique	Pure Al	13 vol.%	91.75%	N/A	4.23 × 10^−4^ mm^3^/m	N/A	[47]
SLM	Pure Al	20 wt.%	97.1%	180 HV_0.1_	4.2 × 10^−5^ mm^3^/(N m)	0.41	This work

## Data Availability

The data presented in this study are available from the corresponding authors upon reasonable request.
